# Mapping of partially overlapping de novo deletions across an autism susceptibility region [*AUTS5*] in two unrelated individuals affected by developmental delays with communication impairment

**DOI:** 10.1002/ajmg.a.32704

**Published:** 2009-03-06

**Authors:** Dianne F Newbury, Pamela C Warburton, Natalie Wilson, Elena Bacchelli, Simona Carone, Janine A Lamb, Elena Maestrini, Emanuela V Volpi, Shehla Mohammed, Gillian Baird, Anthony P Monaco, The International Molecular Genetic Study of Autism Consortium (IMGSAC)

**Affiliations:** 1 Wellcome Trust Centre for Human Genetics, Roosevelt DriveHeadington, Oxford, UK; 2 Guys and St Thomas NHS Foundation TrustLondon, UK; 3 Centre for Integrated Genomic Medical Research, The University of ManchesterManchester, UK; 4 Department of Biology, University of BolognaBologna, Italy; 5 Medical Genetics Laboratory, Policlinico S. Orsola-MalpighiBologna, Italy; 6 http://well.ox.ac.uk/~maestrin/iat.html

**Keywords:** autistic disorder, developmental language disorders, partial monosomy

## Abstract

Autism is a neurodevelopmental disorder characterized by deficits in reciprocal social interaction and communication, and repetitive and stereotyped behaviors and interests. Previous genetic studies of autism have shown evidence of linkage to chromosomes 2q, 3q, 7q, 11p, 16p, and 17q. However, the complexity and heterogeneity of the disorder have limited the success of candidate gene studies. It is estimated that 5% of the autistic population carry structural chromosome abnormalities. This article describes the molecular cytogenetic characterization of two chromosome 2q deletions in unrelated individuals, one of whom lies in the autistic spectrum. Both patients are affected by developmental disorders with language delay and communication difficulties. Previous karyotype analyses described the deletions as [46,XX,del(2)(q24.1q24.2)dn]. Breakpoint refinement by FISH mapping revealed the two deletions to overlap by approximately 1.1Mb of chromosome 2q24.1, a region which contains just one gene—potassium inwardly rectifying channel, subfamily J, member 3 (*KCNJ3*). However, a mutation screen of this gene in 47 autistic probands indicated that coding variants in this gene are unlikely to underlie the linkage between autism and chromosome 2q. Nevertheless, it remains possible that variants in the flanking genes may underlie evidence of linkage at this locus.

## INTRODUCTION

Autism (OMIM 209850) is a neurodevelopmental disorder characterized by a triad of deficits in reciprocal social interaction and communication, and repetitive and stereotyped behaviors and interests [World Health Organisation, 1993; American Psychiatric Association, 1994].

A large-scale UK-based population survey estimated the prevalence of autistic spectrum disorders (ASDs) to be between 0.90% and 1.42% [Baird et al., 2006]. While contemporary studies such as this consistently report a higher incidence than that of traditional investigations, it remains a matter of debate whether this increase represents a genuine trajectory or an improvement in detection and diagnosis.

Family and twin studies reliably indicate the presence of strong genetic factors in the susceptibility to autistic disorder and heritability estimates are generally above 90%. Monozygotic twin concordance rates are significantly higher than those for dizygotic twins and siblings of affected individuals are 20–30 times more likely to develop an ASD than a member of the general population [reviewed by Sykes and Lamb, 2007]. However, it is becoming increasingly clear that, in the majority of cases, the genetics underlying ASDs are likely to be highly complex involving numerous genetic variants at both the sequence and structural level as well as environmental factors [Pickles et al., 1995; Pritchard, 2001; The Autism Genome Project Consortium, 2007].

Over the last decade, several linkage studies have been completed for autism and related disorders [reviewed by Klauck, 2006]. The results of these, alongside other molecular investigations, have meant that almost every chromosome has historically been implicated in the onset of ASDs. However, the abundance of genetic studies has allowed the derivation of a core set of chromosomal regions which appear to be of importance across the broad autistic spectrum. These principal loci are found on chromosomes 2q [Philippe et al., 1999; IMGSAC 2001; Buxbaum et al., 2001; Shao et al., 2002; Morrow et al., 2008], 3q [Auranen et al., 2002; Shao et al., 2002], 7q [CLSA, 1999; IMGSAC, 2001; Liu et al., 2001; Auranen et al., 2002], 11p [The Autism Genome Project Consortium, 2007], 16p [Philippe et al., 1999; IMGSAC, 2001; Lamb et al., 2005], and 17q [IMGSAC, 2001; Yonan et al., 2003; McCauley et al., 2005; Alarcon et al., 2005]. Numerous association studies have been performed within these regions. However, while rare mutations have been reported in some candidate genes, these are often found to affect relatively few autistic individuals and are seldom supported by association trends within larger cohorts. The lack of definitive linkage and association-based results in the autism field are thought to reflect the phenotypic heterogeneity of the disorder and the complexity of the underlying genetic architecture.

An alternative approach, which has proved helpful for other disorders, is the characterization of cytogenetic abnormalities which segregate with disease phenotype. Structural cytogenetic abnormalities are present at a higher rate in autistic cohorts than one would expect to find in the general population [reviewed by Abrahams and Geschwind, 2008]. However, with the exception of maternally derived duplications of a region on chromosome 15q, these abnormalities do not form obvious clusters. In a review of cytogenetic abnormalities in autism, Vorstman et al., 2006 designated chromosomes 2q37, 5p, 7, 11q, 15q, 16q, 17p, 18q, 22q, and Xp as “cytogenetic regions of interest” defined by a minimum of four case reports at the same locus. Recent methodological advances have enabled the screening of the entire genome for both large-scale (cytogenetic) and sub-microscopic (copy number variants—CNVs) deletions and duplications in large autistic populations [Sebat et al., 2007; The Autism Genome Project Consortium, 2007; Marshall et al., 2008; Christian et al., 2008]. In line with the karyotypic findings, each of these studies reported an increased de novo CNV rate in affected individuals (∼7%) above that reported in controls (∼1%). These collaborative efforts have enabled the identification of the first nonsyndromic ASD susceptibility genes including *SHANK3* on chromosome 22q [Durand et al., 2007; Moessner et al., 2007], *NRXN1* on chromosome 2p [Kim et al., 2008], *NLGN3* and *NLGN4* on chromosome X [Jamain et al., 2003; Laumonnier et al., 2004; Yan et al., 2005; Lawson-Yuen et al., 2008] and a microdeletion on chromosome 16p11 [Kumar et al., 2008; Weiss et al., 2008]. However, it is clear that mutations in these genes account for only a small proportion of autistic cases, further supporting the hypothesis that the genetic basis of autism may resemble that of mental retardation, in which the clinical disorder assimilates a variety of distinct rare syndromes that present with similar surface characteristics.

**FIG. 1:**
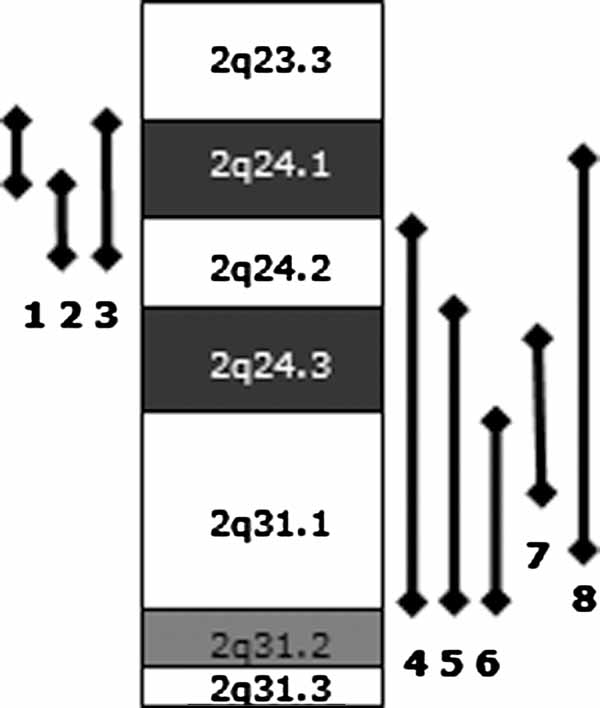
Position of chromosome 2q linkages. Position of current chromosome 2q deletions are shown by the bars on the left. Position of relevant linkage studies are shown on the right. (1) Deletion mapped in patient 1. (2) Deletion mapped in Patient 2. (3) Boundaries of deleted region in Patients 1 and 2. (4) Linkage to autism [Philippe et al., 1999] AUTS5. (5) Linkage to autism [Buxbaum et al., 2001] AUTS5. (6) Linkage to autism [IMGSAC, 2001] AUTS5. (7) Linkage to language impairment [Bartlett et al., 2004]. (8) Linkage to IQ [Posthuma et al., 2005] INTLQ3.

This article describes the molecular cytogenetic characterization of two chromosome 2q deletions in unrelated individuals with developmental delays and language impairment. The difficulties characterized in one of these patients were found to fall within the autistic spectrum (Pervasive Developmental Disorder (PDD)). Routine karyotype analyses described the deletions in these patients as del(2)(q24.1q24.2)de novo. This region is relatively distant to the subtelomeric band commonly deleted in autistic patients [2q37—Lukusa et al., 2004; Falk and Casas, 2007], but lies in a region which has repeatedly shown linkage to autism [*AUTS5*—Philippe et al., 1999; IMGSAC, 2001; Buxbaum et al., 2001; Shao et al., 2002] and has also been implicated to play a role in language impairment [Bartlett et al., 2004] and IQ development [INTLQ3—Posthuma et al., 2005] (Fig. 1) both of which are relevant to the patients described here. By means of fluorescence in situ hybridization (FISH) mapping with bacterial artificial chromosomes (BAC) clone probes along the critical chromosomal bands we define the boundaries of both deletions and the region of overlap. The common deleted segment contains a single gene which encodes a potassium channel expressed in the cardiovascular and nervous systems. We go on to sequence the coding regions of this gene in 47 unrelated autistic probands.

It is hoped that cumulative evidence across studies such as this will aid the search for autism susceptibility genes by allowing the refinement of the large chromosomal regions typically identified by linkage studies.

## MATERIALS AND METHODS

### Clinical Reports

#### Patient 1

This British 12-year-old girl was originally referred because of a mild developmental delay particularly affecting her speech and language. She also had recurrent infections, failure to thrive and short stature.

She is the middle of three children born at term weighing 2.9 kg. Both her siblings were well and there was no contributory family history. The pregnancy was largely uneventful but she was noted to be small for dates. Her growth remained slow in the neonatal period although there were no particular feeding difficulties. She had an operation for bilateral inguinal hernia at 8 weeks and was extensively investigated for her failure to thrive (celiac screen, thyroid function tests and a cystic fibrosis screen were all normal). She was prone to recurrent respiratory tract infections and was found to have a mildly low IGA level. At 18 months she was noted to have a soft ejection systolic murmur which was investigated and proved to be a functional murmur with a structurally normal heart. Subsequently, at the age of 13 years, Patent Ductus Arteriosus (PDA) was diagnosed and surgically corrected. She was also noted to have an apparent convergent squint which remains under review. A formal assessment showed her to have no manifest squint with normal refraction and optic discs. At age 10 years she had a poplideal bursa cyst surgically removed from her leg.

At 8 months, her mother was concerned about her hearing as she was not babbling. The tympanograms were flat. She was due to have grommets inserted when the situation improved and she eventually passed a hearing test.

Her motor milestones were normal: she sat at 6 months, walked at 14 months and had some babble at the age of a year. She was noted to have very few single words by 18 months and concerns regarding her poor speech and slow language acquisition remained.

**FIG. 2:**
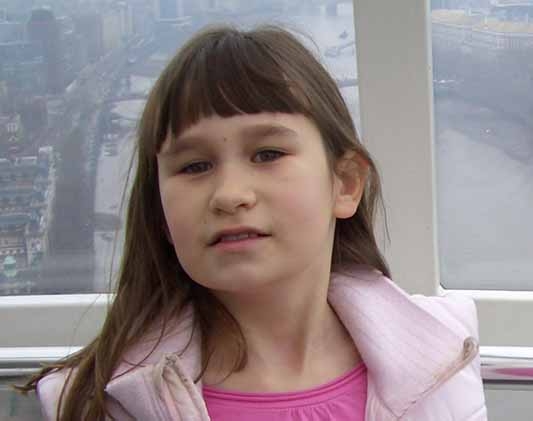
Patient 1. [Color figure can be viewed in the online issue, which is available at www.interscience.wiley.com.]

On examination, she was a petite child with growth parameters between the 2nd and 9th centiles. She was not overtly dysmorphic but had a few distinctive features with slightly deep set eyes, narrow palpebral fissures, a small mouth and somewhat prominent low-set ears (Fig. 2).

Assessment aged 6 years, at a time of continuing concern about communicative competence, with WISC 3 and CELF3 showed particular problems with all language based tasks, receptive score 70, expressive 59 but her performance and verbal IQ were 80 and 73 respectively (all tests mean 100 SD 15). The clinical finding was of “mainly expressive language difficulties but other problems that will affect all aspects of increasingly abstract reasoning and thinking.” She also showed over-anxiety, social anxiety, sensory sensitivities and some rigidity of behaviors.

Later assessment (at age 12 years) using the WISC showed global learning difficulties (FSIQ64), CELF language assessment was in line with the IQ both receptively and expressively. Socially she was immature, in line with IQ and had no autism or ASD clinically or on ADOS.

#### Patient 2

This British girl, of Chinese parentage, was referred at the age of 6½ years with a diagnosis of pervasive developmental disorder and attention deficit difficulties.

She was the second child of unrelated parents. An older sister and younger brother had no learning or behavioral problems. The mother had a nephew who had delayed speech until the age of 4 years but his subsequent development had been normal.

She was born at term following an uneventful pregnancy and delivery. There were no neonatal problems. The parents were first concerned about her development at the age of 18 months. She did not walk independently until 18 months but her language development and communication were significantly more delayed. She started to use words just before the age of 3 years and was assessed to have a complex developmental learning problem with considerable attention deficit which required treatment. She had some obsessive traits and had always been a poor sleeper. In retrospect, there were concerns from the age of 6 months onwards as she showed less eye contact and was poor at play compared to her siblings. Her parents also felt that she had shown slower acquisition of social maturity, sense of danger and that her behavior was sometimes inappropriate compared to her peers.

On examination she was a pleasant and friendly girl who at times could be socially disinhibited. There was no dysmorphism and growth parameters were on the 25th centile. Detailed psychometric assessment showed significant global difficulties in cognition, language and adaptive functioning. Her strengths were felt to be in the areas of understanding and manipulating visually presented material.

When last reviewed at the age of 12 years she had shown considerable progress with her speech and was using some sign language. She was diagnosed to have bi-polar disorder with some psychotic features for which she was receiving appropriate support and treatment.

Both patients were assessed by a single clinician who noted the existence of shared developmental delays and severe expressive communication deficits between individuals.

#### Fluorescence in situ hybridization (FISH)

Lymphoblastoid cell lines were established at the European Collection of Cell Cultures (ECACC) for each patient and used to produce metaphase chromosome spreads fixed on slides. The cells were cultured in RPMI-1640 supplemented with 10% fetal bovine serum (FBS) and 1% l-Glutamine (Sigma,www.sigmaaldrich.com) at 37°C in a 5% CO_2_ incubator. One hour before harvesting the cells were treated with Colcemid (Invitrogen, www.invitrogen.com) at a final concentration of 0.2 µg/ml. They were then resuspended in prewarmed (to 37°C) hypotonic solution (0.0075 M KCl) for 5 min and fixed in three changes of 3:1 methanol: acetic acid. Slides were prepared following standard procedures and aged at −20°C.

BAC cultures were grown overnight and DNA extracted from individual clones by a standard miniprep procedure. DNA was labelled by nick translation (Vysis kit) using either Biotin-16-dUTP or Digoxigenin-11-dUTP (Roche, www.roche-applied-science.com). This labelled BAC DNA was ethanol precipitated in a mix of Salmon testis DNA (Gibco BRL), *Escherichia coli* tRNA (Roche, www.roche-applied-science.com) and 3 M sodium acetate. They were then dried on a heating block with a 50× excess of Human Cot-1 DNA (Gibco) to block repetitive sequences and resuspended in hybridization solution (50% formamide, 10% dextran sulfate, 2× SSC). Denatured probes were pipetted onto the prepared slides and incubated overnight to allow hybridization. The slides were denatured in 70% formamide, quenched in 2× SSC and then dehydrated in an ethanol series. Following hybridization, the slides were washed in 50% formamide and 2× SSC. The Biotinylated probes were detected with Texas Red-conjugated Streptavidin (Invitrogen, www.invitrogen.com), followed by a layer of Biotinylated anti-streptavidin (Vector Laboratories, www.vectorlabs.com) and a final layer of Texas Red-conjugated Streptavidin. The Digoxigenin probes were detected using Mouse anti-Digoxigenin antibody (Roche) and Goat anti-Mouse Alexa-488 (Molecular Probes). The slides were mounted with Vectashield (Vector Laboratories) containing 4′,6-diamidino-2-phenylindole (DAPI) for chromosome counterstaining. The slides were examined using an Olympus BX-51 epifluorescence microscope coupled to a Sensys charge-coupled device (CCD) camera (Photometrics, www.photomet.com). A minimum of 100 nuclei were analyzed for each hybridization experiment. Texas Red, Alexa-488 and DAPI fluorescence images were taken as separate grey-scale images using specific filter combinations and then pseudocoloured and merged using the software package Genus (Applied Imaging International, www.genetix.com).

A BAC tiling path was identified from the UCSC genome browser (http://genome.ucsc.edu/) for the region predicted to contain the deletions. All clones were chosen from the RPCI-11 library [Osoegawa et al., 2001] and obtained from the BACPAC resource center (BPRC) [http://bacpac.chori.org/] as bacterial LB agar stab cultures. The BAC probes used for in situ hybridization are listed in Table I. As the deletion intervals were narrowed, clone pairs were selected independently for each patient and therefore, not every clone was hybridized to both patients.

**TABLE I:** BAC Clones Hybridized to Deletion Patients 1 and 2

Clone	Band	Start	Finish	Patient 1	Patient 2
RP11-185M22	2q23.3	152,371,127	152,550,162	Not deleted	Not deleted
RP11-235N13	2q23.3	152,610,486	152,787,342	Not deleted	
RP11-352J13*	2q23.3	152,777,784	152,967,154	Not deleted	
RP11-173H9*	2q23.3	152,874,124	153,062,670	Deleted	
RP11-17E6	2q23.3	153,090,115	153,243,694	Deleted	Not deleted
RP11-11C17	2q23.3	153,527,697	153,682,796	Deleted	Not deleted
RP11-44N6	2q24.1	154,569,776	154,710,240	Deleted	Not deleted
RP11-79B5	2q24.1	155,284,349	155,457,867	Deleted	Not deleted
RP11-621K10*	**2q24.1**	**155,378,302**	**155,561,125**		**Partially deleted**
**RP11-109N20**	**2q24.1**	**155,561,144**	**155,741,920**		**Deleted**
**RP11-1089G12**	**2q24.1**	**155,671,221**	**155,845,226**		**Deleted**
**RP11-191I9**	**2q24.1**	**155,991,304**	**155,991,738**	**Deleted**	**Deleted**
**RP11-631C11**	**2q24.1**	**155,993,432**	**156,156,880**	**Deleted**	
**RP11-1084C19**	**2q24.1**	**156,144,997**	**156,334,748**	**Deleted**	
RP11-183M18*	**2q24.1**	**156,306,492**	**156,447,588**	**Deleted**	
RP11-637C13*	2q24.1	156,364,263	156,545,772	Not deleted	
*RP11-881C12*	2q24.1	156,527,066	156,717,022	Not deleted	
*RP11-605B16*	2q24.1	156,763,265	156,938,344	Not deleted	Deleted
*RP11-608N6*	2q24.1	157,726,618	157,912,914	Not deleted	Deleted
*RP11-383I5*	2q24.1	157,897,634	158,065,644	Not deleted	Deleted
*RP11-91K6*	2q24.2	159,560,745	159,715,538	Not deleted	Deleted
RP11-292A10*	2q24.2	159,680,594	159,834,459		Partially deleted
RP11-357L2	2q24.2	160,186,041	160,419,283		Not deleted
RP11-615B17	2q24.2	160,419,278	160,629,949		Not deleted

Start and finish values are based on NCBI build 36, UCSC March 2006 assembly.

Underlined clones are deleted in Patient 1, italicized clones are deleted in Patient 2 and clones in bold are deleted in both cases.

Critical BACs, which define the deletion boundaries, are marked with an asterisk (*).

#### Mutation screen

The coding regions of *KCNJ3* were sequenced in 47 unrelated autistic subjects selected from the IMGSAC families that contributed to the linkage peak on chromosome 2q. This strategy selects for cases that are more likely to carry etiological variants at this locus compared to a random patient sample. The ascertainment of these families and the DNA collection procedures have been described in detail elsewhere [IMGSAC, 2001]. DNA from 44 of these IMGSAC patients have previously been characterized on Affymetrix 10K microarrays [The Autism Genome Project Consortium, 2007]. This investigation did not identify any CNVs that segregated with the incidence of autism in these particular cases.

Sequence data were downloaded from NCBI and used to identify intron-exon boundaries. PCR primers flanking these boundaries were designed using the Primer 3 program. For exons greater than 500 bp in size, overlapping PCR products were designed. In total, seven fragments were required to cover the 2.9 kb of genomic sequence. Mutations were identified by direct sequencing of each fragment. Sequencing was performed with BigDye terminator mix on an ABI 3730 and variants were identified using Sequence Navigator. Primer sequences and PCR conditions are available from authors on request.

## RESULTS

In FISH experiments to metaphase chromosome spreads, 18 BAC clones were hybridized to Patient 1, and 16 clones to Patient 2 (Table I).

In Patient 1, the proximal deletion breakpoint was found to lie between the contiguous clones RP11-352J13 (not deleted) and RP11-173H9 (deleted) and the distal deletion breakpoint was found to lie between the neighboring clones RP11-183M18 (deleted) and RP11-637C13 (not deleted) (Table I). This deletion spans approximately 3.6 Mb across 2q23.3–2q24.1 and contains six genes (*FMNL2*–*KCNJ3*—Table II).

**TABLE II:** RefSeq Genes Across the Chromosome 2q23–2q24 Region

Gene	RefSeq name	OMIM no.	Start	Finish	Gene deleted in patient 1?	Gene deleted in patient 2?
*ARL5A*	ADP-ribosylation factor-like 5A	608960	152,365,725	152,393,255	No	No
*CACNB4*	Calcium channel, voltage-dependent, beta 4	601949	152,402,386	152,663,790	No	No
*STAM2*	Signal transducing adaptor molecule 2	606244	152,681,560	152,740,752	No	No
*FMNL2*	Formin-like 2		152,899,996	153,214,594	Yes	No
*PRPF40A*	Formin binding protein 3		153,216,352	153,282,221	Yes	No
*ARL6IP6*	ADP-ribosylation-like factor 6 interacting		153,283,375	153,325,669	Yes	No
*RPRM*	Reprimo, TP53 dependant G2 arrest mediator	612171	154,042,097	154,043,568	Yes	No
*GALNT13*	UDP-*N*-acetyl-D-galactosamine: polypeptide *N*-acetylgalactosaminyltransferase 13	608369	154,436,671	155,018,735	Yes	No
*KCNJ3*	Potassium inwardly rectifying channel, subfamily J, member 3	601534	155,263,338	155,421,260	Yes	Partially
*NR4A2*	Nuclear receptor subfamily 4, group A, member 2	601828	156,889,189	156,897,533	No	Yes
*GPD2*	Glycerol-3-phosphate dehydrogenase 3	138430	157,000,210	157,151,161	No	Yes
*GALNT5*	UDP-*N*-acetyl-alpha-D-galactosamine:polypeptide		157,822,585	157,876,159	No	Yes
*ERMN*	Ermin ERM-like protein isoform a	610072	157,883,370	157,892,392	No	Yes
*PSCDBP*	Pleckstrin homology, Sec7 and coiled-coil	604448	157,979,376	158,008,850	No	Yes
*ACVR1C*	Activin A receptor, type IC	608981	158,091,524	158,193,645	No	Yes
*ACVR1*	Activin A type I receptor precursor	102576	158,301,204	158,440,620	No	Yes
*UPP2*	Uridine phosphorylase 2		158,559,936	158,700,724	No	Yes
*CCDC148*	Coiled-coil domain containing 148		158,736,723	159,021,460	No	Yes
*PKP4*	Plakophilin 4	604276	159,021,721	159,246,186	No	Yes
*DAPL1*	Death associated protein-like 1		159,360,086	159,380,742	No	Yes
*TANC1*	TPR domain, ankyrin-repeat	611397	159,533,391	159,797,416	No	Yes
*WDSUB1*	WD repeat, SAM and U-box domain containing 1		159,800,549	159,851,482	No	Partially
*BAZ2B*	Bromodomain adjacent to zinc finger domain, 2B	605683	159,883,735	160,181,305	No	No
*MARCH7*	Axotrophin		160,277,255	160,333,330	No	No
*CD302*	CD302 antigen		160,333,609	160,362,999	No	No
*LY75*	Lymphocyte antigen 75	604524	160,368,113	160,469,508	No	No

Start and finish values and gene positions are based on NCBI build 36, UCSC March 2006 assembly and taken from the “RefSeq Gene” track of UCSC. Where multiple transcripts are possible, the maximum boundaries have been given for the transcript start and finish positions. No microRNas have been reported across this region (http://microrna.sanger.ac.uk/sequences/index.shtml).

In Patient 2, both RP11-621K10 and RP11-292A10 were found to be partially deleted and therefore represent the boundaries of the deletion in this individual (Table I). This deletion spans approximately 4.5 Mb across 2q24.1–2q24.2 and contains 14 genes (*KCNJ3*–*WDSUB1*—Table II).

**FIG. 3:**
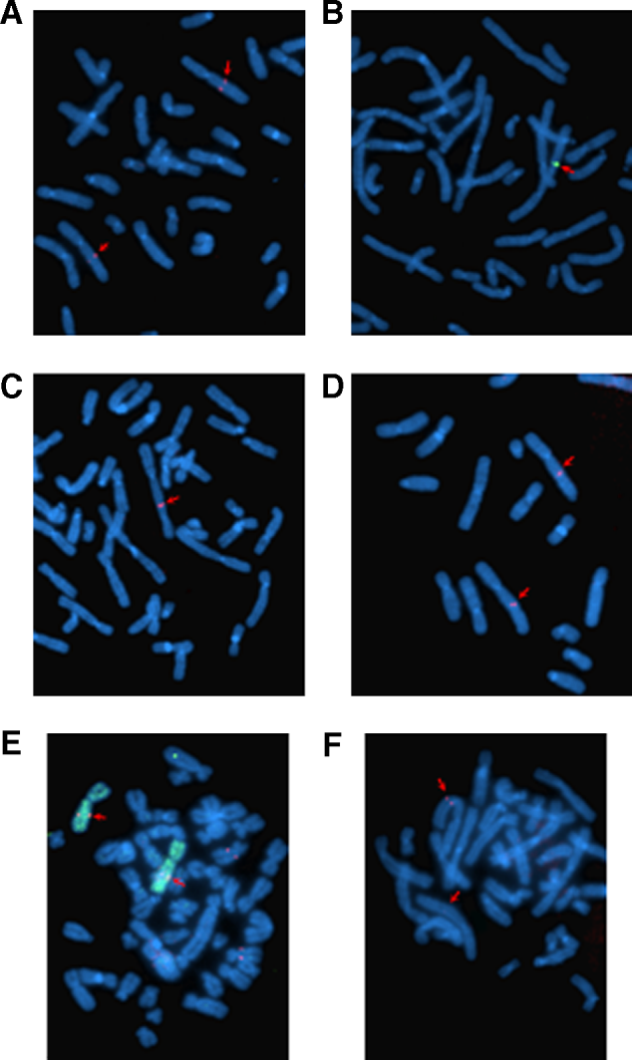
Deletion mapping by FISH on metaphase chromosomes: critical clones. For each image, the position of the hybridization sites of the relevant clones is marked by an arrow. Images **A**–**D** are from Patient 1. Images **E** and **F** are from Patient 2. Images A and B show the proximal boundaries of the deletion in Patient 1. Images C and D show the distal boundaries of the deletion in Patient 1. Images E and F show partially deleted clones spanning the deletion boundaries in Patient 2. A: RP11-352J13 hybridizes to both copies of chromosome 2. B: RP11-173H9 is deleted on one copy hybridizes to both copies of chromosome 2. C: RP11-183M18 hybridizes to a single copy of chromosome 2. D: RP11-637C13 hybridises to both copies of chromosome 2. E: RP11-621K10 shows a bright signal on one copy of chromosome 2, and a weaker signal on its homologue indicating that it is partially deleted on one copy. Note that this clone also hybridizes to another chromosome and this image therefore includes a chromosome 2 paint allowing the identification of the significant hybridizations. F: RP11-292A10 shows a bright signal on one copy of chromosome 2, and a weaker signal on its homologue indicating that it is partially deleted on one copy.

FISH images of the critical clones on metaphase chromosome spreads are shown in Figure 3.

The two deletions overlap by approximately 1.1Mb of 2q24.1 (155,378,302–156,447,588) and this region contains just one gene—potassium inwardly rectifying channel, subfamily J, member 3 (*KCNJ3*) (OMIM 601534) (Table II). While the deletion boundaries cannot be absolutely defined by FISH analysis, it appears that the deletion in Patient 1 spans the entire *KCNJ3* gene whilst only the 3′ end of this gene is deleted in Patient 2 (BAC clone RP11-621K10).

Mutation screening of the coding regions and putative functional sequences of *KCNJ3* revealed seven sequence changes within the IMGSAC sample: Four synonymous coding changes, two variants in the 5′UTR region and one very rare variant in the promoter region (Table III). Two of the coding variants (silent H346 and silent S197) were previously characterized polymorphisms within the dbSNP database (rs17642086 and rs3111033, respectively) and no significant difference in allele frequencies was identified between the autism and reported Perlegen and HapMap CEPH samples. The other two synonymous variants were both very rare. Table III gives full details of all changes found.

**TABLE III:** Coding Changes Found in the KCNJ3 Gene

Location in gene	Chromosome position	DNA variant	Type of mutation	MAF autism^a^	dbSNP ID
Promoter	155,262,749	C/T		0.01	
Exon1	155,263,336	G/A	5′UTR	0.04	rs3111034
Exon1	155,263,446-7	—/C	5′UTR	0.35	rs5835535
Exon1	155,263,611	C/T	Silent (P26)	0.01	
Exon1	155,264,124	C/T	Silent (S197)	0.02	rs3111033
Exon3	155,419,603	T/C	Silent (H346)	0.33	rs17642086
Exon3	155,420,059	T/C	Silent (D498)	0.01	

Chromosome positions are based on NCBI build 36, UCSC March 2006 assembly.

a Minor allele freq in 47 IMGSAC individuals with autism.

As part of a high-density SNP genotyping and association study carried out across the chromosome 2q24–q32 region, we have tested 42 tag SNPs, selected using data from the HapMap project to capture common genetic variation within *KCNJ3* genomic regions. The 42 tag SNPs have been genotyped in a sample of 126 parents–child trios selected from IMGSAC multiplex families to be linked to this region of chromosome 2 and 188 sex-matched random European controls (ECACC). None of the SNPs provided significant evidence of association using either family-based or case–control analysis (data not shown, manuscript in preparation).

Other genes surrounding the common deletion region include UDP-*N*-acetyl-D-galactosamine: polypeptide N-acetylgalactosaminyltransferase 13 (*GALNT13*—MIM608639), which is deleted in Patient 1 and lies approximately 350 kb from the deletion in Patient 2, and nuclear receptor subfamily 4, group A, member 2 (*NR4A2*—MIM601828) which is deleted in Patient 2 and lies approximately 450 kb distal to the deletion in Patient 1 (Table II).

The 7 Mb spanned by the pair of deletions (chromosome position 152,874,124–159,834,459), is covered by a single contig (NT_005403) and contains 19 known genes (*FMNL2*–*WDSUB1*—Table II). Of these 19 genes, all but one (*GALNT5*) show some level of expression in the brain and therefore most may be argued to represent good candidate genes for ASDs and related neurodevelopmental disorders.

## DISCUSSION

This study describes the mapping of two partially overlapping, de novo, deletions involving chromosome 2q24 in unrelated individuals affected by developmental delay, language impairment and social difficulties. These deletions map within or close to regions of chromosome 2q identified by linkage studies as candidate regions for autism (*AUTS5*), language impairment and IQ (Fig. 1). Furthermore, this is one of only two regions which have achieved genomewide significance for autistic disorder [IMGSAC, 2001]. However, the regions identified by linkage studies are large and the selection and screening of candidate genes can often prove to be a perplexing task. Two studies have found positive association to microsatellite markers on chromosome 2q [Romano et al., 2005; Lauritsen et al., 2006], however, both of these investigations were performed using relatively isolated populations and the associations described map distal to the region involved in our patients (2q31.1). A few studies have targeted candidate genes on chromosome 2q [Bacchelli et al., 2003; Rabionet et al., 2004; Hamilton et al., 2005; Blasi et al., 2006], but only one has yielded any positive results. Bacchelli et al. 2003 screened the coding regions of nine genes within 2q24.2–2q31.3 and performed an association analysis using SNPs from this region. They identified four rare nonsynonymous mutations within the *cAMP-GEFII* gene on chromosome 2q31.1 (OMIM 606058) which segregated with the autistic phenotype in five of their 169 families. However, Bacchelli et al., caution that the frequency of mutation could not account for the linkage signal on chromosome 2q and the findings of this study have yet to be replicated. No autism study to date has specifically investigated any of the genes contained within the deletions reported here. It is therefore hoped that the detailed characterization of chromosome abnormalities in patients with relevant phenotypes, such as those reported here, may aid the mapping of susceptibility genes by the reduction of candidate linkage regions.

The overlap between the two deletions is relatively small (1.1 Mb) and contains only one gene—*KCNJ3*. *KCNJ3* encodes a G-protein-gated inward-rectifier potassium channel (GIRK), which imports potassium into a cell at a much higher rate than it exports it. There are four known GIRK subunits and these interact in various combinations to form functional heterodimeric channels. GIRKs are involved in a variety of cellular processes including cell excitability, heart rate, vascular tone and insulin release. In the brain, GIRK channels control neuronal excitability and plasticity [Mark and Herlitze, 2000]. The gene is 158 kb in length and contains 3 exons encoding a 2.9 kb transcript and a 501 amino acid protein [Schoots et al., 1996]. The database of Genomic Variants [Iafrate et al., 2004] reports no known CNVs within this gene. A SNP in the *KCNJ3* gene has been reported to be associated with idiopathic generalized epilepsy [rs17642086 in Table III, *P* = 0.0097, Chioza et al., 2002]. Specifically, association at this SNP was strongest in the subgroup of probands with absence seizures. Although the prevalence of epilepsy in children with autism is significantly increased above that of the general population [Levisohn, 2007], neither of the patients described in this article have a history of seizure activity. Since they have both passed the mean age of seizure onset in the Chioza sample (11.7 years), it is unlikely that the deletion of this gene has caused an epilepsy/autism type syndrome in these patients. Sequence analysis of this gene in 47 autistic probands failed to identify any mutations which suggest a role for *KCNJ3* in ASD susceptibility.

However, if we consider the possibility of positional effects, which have been reported to occur up to 1 Mb away from translocation breakpoints [Pfeifer et al., 1999], the “critical region” identified by the two patients studied here may be expanded to approximately 3 Mb. This extended area contains three additional candidate genes—UDP-*N*-acetyl-alpha-D-galactosamine:polypeptide *N*-acetylgalactosaminyltransferase 13 (*GALNT13*), nuclear receptor subfamily 4, group A, member 2 (*NR4A2*) and glycerol-3-phosphate dehydrogenase 2 (*GPD2*) (OMIM 138430) (Table II). *NR4A2*, which is deleted in Patient 2 and lies approximately 450 kb distal to the deletion in Patient 1, is essential for the differentiation of the nigral dopaminergic neurons. Interestingly, this gene has been implicated in a range of neurological disorders including heroin addiction [Nielsen et al., 2008], antisocial behavior in women [Prichard et al., 2007], schizophrenia [Rojas et al., 2007] and Parkinson Disease [Xu et al., 2002; Le et al., 2003]. *GALNT13*, which is deleted in Patient 1 and lies approximately 350 kb proximal of the deletion in Patient 2, shows a brain specific expression pattern, which is particularly high in the foetal brain. It is involved in the O-linked glycosylation of epithelial glycoproteins [Zhang et al., 2003]. Two small variants have been reported to exist in introns 1 and 2 of this gene [de Smith et al., 2007; Wong et al., 2007] but no variations have been reported that affect the coding sequences. Glycerol-3-phosphate dehydrogenase 2 (*GPD2*) is involved in glycerol metabolism and has been implicated in type II diabetes [Novials et al., 1997]. All four of the genes described above are expressed at some level in the brain and so should therefore be considered as possible candidate genes for the disorder in these two patients.

The Autism Chromosome Rearrangement Database [ARCD—http://projects.tcag.ca/autism/, Marshall et al., 2008] describes only one autistic structural variant in the chromosome 2q24.1–2q24.2 region. This patient has a chromosome 2 to chromosome 9 translocation (46,XY,t(2;9)(q24.2;p24)) and is described as having autism, mental retardation, speech defect, scaphocephaly/dolichocephaly, behavior disorder, hyperactivity, psychosis and upslanting palpebral fissures (MCN patient 19940001-113). In addition, in a review article, Lauritsen et al. 1999 includes a patient with moderate mental retardation and autistic features and a complex rearrangement involving a large portion of chromosome 2q (46,XY,t(1;2;13)(p21.2;q24.2-q36.2;q14.3)de novo). In both the above cases, however, no further details regarding the breakpoints could be determined.

There has been much recent debate regarding the relationship between the characteristic triad of deficits seen in autism (reciprocal social interaction, communication, and repetitive and stereotyped behaviors and interests) and the overlaps between these impairments and those seen in other developmental disorders such as Specific Language Impairment (SLI) and mental retardation (MR). Although the majority of autistic individuals display deficits in all three of the triad areas, there remain individuals who fail to meet criteria for one or more of the triad definitions. Furthermore, even in those individuals who do carry the full triad of deficits, the exact nature of the impairments varies considerably both between individuals and over time. Thus, in light of emerging evidence that identified genetic variants play a role in a relatively rare number of cases with characteristic deficits, there is growing support for a degree of genetic separation between deficit areas and for the existence of shared genetic risk variants between developmental disorders [Bailey et al., 1998]. Under such a hypothesis, the surface presentation of the disorder is highly dependent upon additional modifier components which may be genetic and/or environmental in nature. Thus although only one of the patients investigated in this article had a positive diagnosis of ASD, the degree of overlap between their impairments and the presence of an overlapping chromosome abnormality was sufficient to suggest a shared etiology. Furthermore, given the presence of ASD in one of the cases and the continued links between the deleted region and ASDs in the wider population, it may be argued that the genes affected by the deletions reported here should also be regarded as possible candidates in idiopathic cases of autistic disorder. Advances in microarray technology mean that it would now be possible to perform genome-wide screens for ancillary, sub-microscopic, rearrangements (CNVs) or additional shared genetic features in these patients. Such a step would enable the evaluation of the described deletion in the context of the whole genome and may therefore provide valuable information regarding the relationship between shared genetic and surface features in these two individuals.

Previous investigations have demonstrated the value of multiple clustered chromosome abnormalities in the search for genes underlying linkage signals and it would therefore be of interest to also screen the three genes surrounding the deletion in a larger autistic cohort. Recent evidence suggests that the parallel use of complementary genetic approaches will ultimately enable the identification of genes which predispose individuals to the development of autistic traits, and it is hoped that this will promote a better understanding of the biological basis of these disorders.
